# Characterizing Plasmids in Bacteria Species Relevant to Urinary Health

**DOI:** 10.1128/spectrum.00942-21

**Published:** 2021-12-22

**Authors:** Cesar Montelongo Hernandez, Catherine Putonti, Alan J. Wolfe

**Affiliations:** a Department of Microbiology and Immunology, Stritch School of Medicine, Loyola University Chicago, Maywood, Illinois, USA; b Bioinformatics Program, Loyola University Chicago, Chicago, Illinois, USA; c Department of Biology, Loyola University Chicago, Chicago, Illinois, USA; Dublin City University

**Keywords:** plasmids, urinary tract, microbiota, incompatibility group, rep, genome, microbial, Gram-negative bacteria, Gram-positive bacteria, urinary tract infection

## Abstract

The urinary tract has a microbial community (the urinary microbiota or urobiota) that has been associated with human health. Whole genome sequencing of bacteria is a powerful tool, allowing investigation of the genomic content of the urobiota, also called the urinary microbiome (urobiome). Bacterial plasmids are a significant component of the urobiome yet are understudied. Because plasmids can be vectors and reservoirs for clinically relevant traits, they are important for urobiota dynamics and thus may have relevance to urinary health. In this project, we sought plasmids in 11 clinically relevant urinary species: Aerococcus urinae, Corynebacterium amycolatum, Enterococcus faecalis, Escherichia coli, Gardnerella vaginalis, Klebsiella pneumoniae, Lactobacillus gasseri, Lactobacillus jensenii, Staphylococcus epidermidis, Streptococcus anginosus, and Streptococcus mitis. We found evidence of plasmids in E. faecalis, E. coli, K. pneumoniae, S. epidermidis, and S. anginosus but insufficient evidence in other species sequenced thus far. Some identified plasmidic assemblies were predicted to have putative virulence and/or antibiotic resistance genes, although the majority of their annotated coding regions were of unknown predicted function. In this study, we report on plasmids from urinary species as a first step to understanding the role of plasmids in the bacterial urobiota.

**IMPORTANCE** The microbial community of the urinary tract (urobiota) has been associated with human health. Whole genome sequencing of bacteria permits examination of urobiota genomes, including plasmids. Because plasmids are vectors and reservoirs for clinically relevant traits, they are important for urobiota dynamics and thus may have relevance to urinary health. Currently, urobiota plasmids are understudied. Here, we sought plasmids in 11 clinically relevant urinary species. We found evidence of plasmids in E. faecalis, E. coli, K. pneumoniae, S. epidermidis, and S. anginosus but insufficient evidence in the other 6 species. We identified putative virulence and/or antibiotic resistance genes in some of the plasmidic assemblies, but most of their annotated coding regions were of unknown function. This is a first step to understanding the role of plasmids in the bacterial urobiota.

## INTRODUCTION

##  

Until recently, urine was assumed to be sterile (1, 2). It is now confirmed that resident microbial communities (microbiota) exist even in asymptomatic individuals and that specific species are associated with urinary conditions, such as overactive bladder (OAB), urge urinary incontinence (UUI), and urinary tract infection (UTI) (3–6). Multiple bacterial species make up the urinary microbiota (urobiota), with species composition, cell count, and population dynamics linked to urinary conditions or lack of symptoms (7–9). The bacterial urinary microbiome (urobiome) includes not just the sequence of a bacteria’s chromosome but also mobile genetic elements, such as prophages and plasmids (10–12). Plasmids can serve as transmissible reservoirs for clinically relevant traits, such as antibiotic resistance, virulence, and fitness genes (13–15). Plasmids can be genetically heterogenous; therefore, conserved replicon genes such as those involved in replication (*rep*) or incompatibility (*inc*) are used for profiling (16–19). Bacterial plasmids in the urinary tract are understudied yet could be relevant to urinary health and clinical management, as they are in other microbiota niches (20–22). For example, an urgent question in urinary research is why some bacteria may be present in both asymptomatic people and those with lower urinary tract symptoms (LUTS); given their role in pathogenicity, plasmids are a logical target to study in this context. In this project, we analyzed the presence and properties of plasmids in 11 clinically relevant urinary species: A. urinae, C. amycolatum, E. faecalis, E. coli, G. vaginalis, K. pneumoniae, L. gasseri, L. jensenii, S. epidermidis, S. anginosus, and S. mitis. These commensals, pathogens, and opportunistic pathogens are representative of the diverse taxa commonly detected in the urinary tract (3, 23–31). A. urinae, C. amycolatum, E. coli, E. faecalis, K. pneumoniae, S. epidermidis, S. anginosus, and S. mitis have been associated with UTI, although all these species can also be present in asymptomatic individuals (3, 23, 24, 27, 29–32). In particular, E. coli and K. pneumoniae are the species most commonly associated with UTI (33–35). *Gardnerella* species can be present in the bladders of women asymptomatic for urinary conditions, although one study found *Gardnerella* in a larger proportion of participants with OAB (23). *Lactobacillus* species are generally thought to be commensal bacteria, as appears to be the case for L. jensenii, which has been shown to have antibacterial activity against E. coli (28, 36). In contrast, L. gasseri is more frequently detected in women with UUI (23), but the function of this *Lactobacillus* species is unknown.

Current whole genome sequencing (WGS) studies of urinary tract bacteria species have focused on their overall genomic content, with less emphasis on any plasmids they might contain (26, 30, 37–39). E. coli is the species most commonly associated with UTI (33–35), and its plasmids in the urinary tract are some of the best-studied relative to other urinary species (40–42). F plasmids, often associated with *incF* loci, are an especially important plasmid type in Enterobacteriaceae, the family that includes both E. coli and K. pneumoniae. The F plasmid is considered to be widespread and able to frequently transmit both antibiotic resistance and virulence genes (15, 20, 43). IncF plasmids usually are considered to have a narrow host range, only being transferred and maintained in Enterobacteriaceae (44, 45). Plasmids have been identified in in K. pneumoniae isolates from patients with UTI and have been proposed as a fingerprint to identify the origin of infection (46, 47).

Plasmids in Gram-positive organisms are significantly less characterized, although progress has been made in clinically relevant bacteria, such as Staphylococcus species (17, 48, 49). Staphylococci plasmids are relevant to antibiotic resistance and virulence, and gene content from these can be homologous to other Gram-positive species (17, 49, 50). Plasmids have been profiled in Staphylococcus saprophyticus isolates from women with UTI (51, 52). There is much less known about the role that plasmids from other species play in the urinary tract, but we can make inferences from our broader understanding of their plasmid biology. Plasmids in *Enterococcus* are credited for its infamous multi-drug resistance (17, 53). Lactobacillus and Streptococcus species have evidence of plasmids but are understudied relative to other species mentioned here (54–57). Finally, very little is known of plasmids in A. urinae and C. amycolatum, even in the broader microbiology literature (58, 59).

In this study, we utilized genomics to (i) identify plasmids in representative strains of clinically relevant urinary species and (ii) profile plasmid-encoded loci relevant to urinary health. We provide evidence of plasmid presence by generating plasmidic assemblies, identifying replicon-associated loci, and analyzing similarity to the plasmid entries in the NCBI nr/nt database. There is evidence of plasmids in 5 of the 11 urinary species tested (E. coli, E. faecalis, K. pneumoniae, S. epidermidis, and S. anginosus), some of which carry classic antibiotic resistance and virulence genes. In addition, most putative plasmids have numerous predicted open reading frames that code for proteins with unknown function, underscoring the need for more detailed studies of plasmids in the urinary microbiota. Our findings may be used to prioritize urinary bacterial species whose plasmids could be profiled sufficiently by WGS, while identifying species that require more in-depth analysis, potentially with a wet lab component for plasmid identification. Plasmid research in the urinary tract is an understudied topic, especially in the context of LUTS, and understanding plasmids in these species could provide insight into the role of bacteria in urinary health.

## RESULTS

First, we analyzed a set of draft genomes from 71 urinary isolates from 11 different species for presence of plasmids, including species with well-studied plasmids (E. coli, E. faecalis, K. pneumoniae, and S. epidermidis) and less well-studied species (A. urinae, C. amycolatum, G. vaginalis, L. gasseri, L. jensenii, S. anginosus, and S. mitis). Most strains were isolated from females (N = 69) with or without lower urinary tract symptoms; full details are listed in Table S1 in the supplemental material. To achieve that goal, we generated plasmidic assemblies from the raw reads of these isolates using plasmidSPAdes, which is optimized for identifying and assembling circular sequences, such as plasmids. Plasmidic assemblies could not be generated from the raw sequence reads for any of the A. urinae, G. vaginalis, and S. mitis isolates, and most of the isolates from C. amycolatum, L. gasseri, and L. jensenii (Table S1). Plasmidic assemblies were generated for the rest of the isolates; we searched these for replicon loci via PlasmidFinder. Five urinary species (E. coli, E. faecalis, K. pneumoniae, S. epidermidis, S. anginosus) were predicted to include replicon (*inc* and *rep*) loci in some of their plasmidic assemblies (Table 1). We used VirulenceFinder and ResFinder to identify virulence and antibiotic resistance genes in the plasmidic assemblies. Most plasmidic assemblies had predicted virulence and/or antibiotic resistance genes (Table 1).

**TABLE 1:** Replicon, virulence, and antibiotic resistance gene profiling of urinary bacteria plasmidic assemblies

Strain that generated plasmidic assembly	Species	Inc/rep hits	Virulence hits	Antibiotic resistance hits
UMB9250	Escherichia coli	IncX1		
UMB1195	Escherichia coli	IncB/O/K/Z	traT	
UMB1284	Escherichia coli	IncFIA, IncFII	traT	Tetracycline
UMB1284_2	Escherichia coli	IncX1		
UMB1180	Escherichia coli	Col440I		
UMB7764	Escherichia coli	IncFIB, IncFII	sitA, MntB_1, mntB_2, mntB_3	Trimethoprim, sulfamethoxazole, amoxicillin, beta-lactam, multi-drug
UMB9246	Escherichia coli	IncFIA, IncFIB	iucC, iutA, sitA, mntB_1, mntB_2, mntB_3	Tetracycline
UMB7780	Enterococcus faecalis		cylA, cylL, cylM	
UMB0843	Enterococcus faecalis		agg	
UMB7783	Klebsiella pneumoniae	IncFII(K)	traT	
UMB8492	Klebsiella pneumoniae			
UMB7779	Klebsiella pneumoniae	IncFII(K)	traT	
UMB8493	Staphylococcus epidermidis			
UMB1227	Staphylococcus epidermidis	rep20		Macrolide, bacitracin
UMB0626	Staphylococcus epidermidis			Macrolide, antiseptic/disinfectant
UMB0593	Staphylococcus epidermidis			
UMB1201	Staphylococcus epidermidis			
UMB9183	Staphylococcus epidermidis	rep7a		Tetracycline
UMB0567	Streptococcus anginosus			
UMB8616	Streptococcus anginosus			

Six of nine E. coli isolates had evidence of at least one predicted plasmidic assembly; one of these had evidence of two. We confirmed the bioinformatic prediction of these plasmids for three of the E. coli strains by Nanopore long-read sequencing, generating hybrid assemblies for the strains (Table 2). The replicons represented in the urinary E. coli plasmidic assemblies were Col440I, IncB/O/K/Z, IncFIA, IncFIB, IncFII, and IncX1 (Table 1). From the seven E. coli plasmidic assemblies, there were virulence genes in four and antibiotic resistance genes in three (Table 1). The plasmidic assembly from UMB7764, predicted to be an F plasmid, had three distinct predicted genes for antibiotic resistance against trimethoprim, sulfamethoxazole, and beta-lactams. Plasmidic assemblies from E. coli strains UMB1195 and UMB1284 were predicted to encode *traT*, a factor that blocks invasion of similar or same plasmids and protects bacteria against some bacteriophages and killing by animal blood serum (60–63). TraT is thus considered a virulence factor. The plasmidic assembly in UMB7764 had predicted metal scavenging genes (including *sitA*, which can bind metals and aid in adhesion) and manganese transporters (*mntB_1*, *mntB_2*, and *mntB_3*). Lastly, the UMB9246 isolate’s plasmidic assembly was predicted to encode *sitA*, as well as the aerobactin synthase *iucC* (involved in iron transport) and *iutA* (which binds aerobactin).

**TABLE 2:** Comparison of predicted plasmidic assemblies from plasmidSPAdes assembly of short-read sequencing and long-read assembly

Strain	Length of short-read assembled plasmid (topology)	Length of long-read assembled plasmid (topology)
UMB1180	4990 (linear)	4863 (circular)
UMB1195	94010 (linear)	85640 (circular)
UMB1284	75565 (linear)	98469 (linear)
UMB1284_2	35646 (linear)	35519 (circular)

All three putative K. pneumoniae plasmids had high query coverage and sequence identity (>90%) to annotated complete plasmid records in GenBank (Table 3). Two K. pneumoniae plasmidic assemblies (UMB7783, UMB7779) had the Klebsiella variant of the *incFII* gene, which is associated with virulence plasmids (Table 1). UMB7783 and UMB7779 were predicted to encode TraT (Table 1).

**TABLE 3:** Alignment of urinary plasmidic assemblies to reference plasmids

Strain	Taxonomy	Urinary plasmid size (bp)	Plasmid hit	NCBI plasmid size (bp)	Sequence query coverage	E value	Per. Ident	Accession	PMID
UMB9250	Escherichia coli	43458	p30155-2	54008	56%	0	98.65%	CP053050.1	32883017
UMB1195	Escherichia coli	94010	p86	86147	81%	0	99.18%	CP023387.1	29102123
UMB1284_1	Escherichia coli	75565	p179-1	122483	85%	0	100.00%	CP041560.1	32042895
UMB1284_2	Escherichia coli	35646	p51008369SK1_C	33826	95%	0	99.95%	CP029976.1	N/A
UMB1180	Escherichia coli	4990	pEcl5-3	4863	100%	0	99.98%	CP047739.1	33122675
UMB7764	Escherichia coli	58200	p1658/97	125491	69%	0	99.81%	AF550679.1	17220406
UMB9246	Escherichia coli	73121	pSCU-313-1	105394	87%	0	99.84%	CP051695.1	32759337
UMB7780	Enterococcus faecalis	64875	p26975_2#26	66716	47%	0	99.91%	LR962696.1	N/A
UMB0843	Enterococcus faecalis	47683	p26975_1#7	56311	77%	0	98.86%	LR961992.1	N/A
UMB7783	Klebsiella pneumoniae	96352	pAR_0096	100759	100%	0	100.00%	CP027614.1	N/A
UMB8492	Klebsiella pneumoniae	99217	pAR_0096	100759	100%	0	99.94%	CP027614.1	N/A
UMB7 779	Klebsiella pneumoniae	102300	pKpn3-L132	150325	80%	0	99.87%	CP040025.1	31665400
UMB8493	Staphylococcus epidermidis	47203	pSE1	51568	42%	0	98.84%	CP066375.1	N/A
UMB1227	Staphylococcus epidermidis	49935	pSP01	76991	52%	0	99.87%	KR230047.1	26472766
UMB0626	Staphylococcus epidermidis	38439	pFDAARGOS_161	21267	58%	0	99.60%	CP014130.1	N/A
UMB0593	Staphylococcus epidermidis	19949	pER01533.3	26684	23%	0	99.90%	CP030674.1	N/A
UMB1201	Staphylococcus epidermidis	47834	pSESURV_p1_0612	51026	32%	0	99.95%	CP043786.1	32004459
UMB9183	Staphylococcus epidermidis	4566	pSEP1	4439	100%	0	99.98%	AP019722.1	N/A
UMB0567	Streptococcus anginosus	6660	pDRPIS7493	4727	32%	0	97.65%	CP002926.1	21994930
UMB8616	Streptococcus anginosus	4935	paSTHERMO	4451	86%	0	97.40%	LR822024.1	N/A

No replicon or antibiotic resistance gene was predicted in the three identified E. faecalis plasmidic assemblies (Table 1). However, the putative plasmid sequence from E. faecalis strain UMB0843 was predicted to encode the virulence gene *agg*, involved in agglutination and adhesion, specifically in the context of promoting content for plasmid conjugation (64). The plasmidic assembly from E. faecalis UMB7780 was predicted to encode genes from the cytolysin operon consisting of the activator (*cylA*) and lysin (*cylL*, *cylM*).

For the six plasmidic assemblies of S. epidermidis, only UMB1227 (*rep20*) and UMB9183 (*rep7A*) had identifiable replicon loci (Table 1). Macrolide resistance was predicted in UMB1227 and UMB0626, while tetracycline resistance was predicted in UMB9183. The two plasmidic assemblies from *S. anginosus* had no predicted virulence or antibiotic resistance genes.

Plasmidic assemblies were compared to entries in the NCBI nr/nt database via BLAST. Those with relatively high homology (over 20% sequence query coverage) to plasmid entries are listed in Table 2, and those with lower homology (less than 20% sequence query coverage) to plasmid entries are in Table S2; plasmidic assemblies with no hits to plasmid entries are not listed. Relative to known plasmids, five of the seven E. coli plasmidic assemblies had a query coverage over 80% and sequence identity over 99%, in addition to a similar sequence length to the reference plasmid entry (Table 3). All three K. pneumoniae plasmidic assemblies had high homology to their respective database records and were estimated to be relatively large (∼100 kbp) (Table 3). One E. faecalis isolate had no evidence of a plasmid (i.e., no plasmidic assembly), but the two other isolates had plasmidic assemblies that had 47% and 77% sequence query coverage, respectively, and over 99% identity to Enterococcus plasmids in the database (Table 3). Six of the nine S. epidermidis isolates had plasmidic assemblies of 20k–50k bases; although their query coverage was in the 30–50% range, their sequence identity was >98% relative to plasmid entries (Table 3). Two of eight S. anginosus isolates had plasmidic assemblies, approximately 50k bases in size, and 32% or 86% sequence query coverage over 97% identity, respectively (Table 3). Some isolates from C. amycolatum and the Lactobacillus species had plasmidic assemblies with low query coverage to plasmid entries in the NCBI nr/nt database (Table S1, Table S2). Raw sequencing reads were mapped to the plasmid sequence in the NCBI database with the highest homology score, which improved the query coverage when comparing urinary isolates to that reference (Table S3).

We annotated and then counted all (open reading frames) ORFs in the plasmidic assemblies; the greatest number of ORFs (N = 241) was in the plasmidic assembly from E. coli UMB1195 (∼94k bp) (Table 4). We calculated the ratio of ORFs with a predicted function to all predicted ORFs (predicted and hypothetical function). The overall percentage of ORFs annotated with an assigned function ranged from zero to 60.71% in all assemblies, with the highest percentages present in plasmidic assemblies from S. epidermidis, E. coli, and K. pneumoniae. On average, in all the plasmidic assemblies, 31.38% of ORFs were annotated with an assigned function.

**TABLE 4:** Summary of annotated content in urinary bacteria plasmidic assemblies

Isolate	Species	Plasmidic assembly size (bp)	Total ORF predicted	ORF annotated with function	ORF annotated as hypothetical	% annotated with function
UMB1180	Escherichia coli	4990	4	0	4	0
UMB1195	Escherichia coli	94010	241	29	212	12.03
UMB1284	Escherichia coli	75565	78	38	40	48.72
UMB1284_2	Escherichia coli	35646	47	11	36	23.4
UMB7764	Escherichia coli	58200	61	35	26	57.38
UMB9246	Escherichia coli	73121	76	40	36	52.63
UMB9250	Escherichia coli	43458	53	15	38	28.3
					avg %	31.78
UMB0843	Enterococcus faecalis	47683	51	6	45	11.76
UMB7780	Enterococcus faecalis	64875	110	37	73	33.63
					avg %	22.7
UMB7779	Klebsiella pneumoniae	102300	108	53	55	49.07
UMB7783	Klebsiella pneumoniae	96352	110	39	71	35.45
UMB8492	Klebsiella pneumoniae	99217	121	40	81	33.06
					avg %	39.19
UMB0567	Streptococcus anginosus	6660	6	0	6	0
UMB8616	Streptococcus anginosus	4935	4	1	3	25
					avg %	12.5
UMB0593	Staphylococcus epidermidis	19949	22	6	16	27.27
UMB0626	Staphylococcus epidermidis	38439	4	1	3	25
UMB1201	Staphylococcus epidermidis	47834	28	17	11	60.71
UMB1227	Staphylococcus epidermidis	49935	44	28	16	63.63
UMB8493	Staphylococcus epidermidis	47203	50	11	39	22
UMB9183	Staphylococcus epidermidis	4566	4	2	2	50
					avg %	41.43
					Total avg %	31.38

Next, we reviewed the plasmidic assembly annotations for genes involved in plasmid biology (transfer, replication, and retention). Only two bacterial species, E. coli and K. pneumoniae, had known conjugation genes in their plasmidic assemblies. Per the Prokka annotation, these consisted of transfer (*tra*) genes or virB-virD4 genes (Table 5). Genes that block plasmid fertility were predicted in E. coli and K. pneumoniae plasmidic assemblies, with *finO* genes present in E. coli UMB7764, E. coli UMB9246, K. pneumoniae UMB7783, and K. pneumoniae UMB8492. E. coli UMB1284 and UMB9246 had a complete module of the toxin-antitoxins (TA) *ccdAB* and *pemIK*, which are involved in plasmid addiction function. E. coli UMB9250 and S. epidermidis UMB8493 were predicted to code the toxin component of a TA, but the antitoxin gene was not annotated by Prokka.

**TABLE 5:** Summary of plasmid-related genes in urinary bacteria plasmidic assemblies

Strain	Species	Plasmid transfer	Plasmid replication	Plasmid retention
UMB9250	Escherichia coli	virB4, ptIE, virB9, virB11, virD4		relE
UMB1195	Escherichia coli		parM, ssb (plasmid)	
UMB1284	Escherichia coli	traD		ccdB, ccdA, pemI, pemK
UMB1284_2	Escherichia coli	virB4, virB8, vir9, vir11		
UMB1180	Escherichia coli			
UMB7764	Escherichia coli	finO, traD, traI	ssb (plasmid), repB, vapB	
UMB9246	Escherichia coli	finO	repB	pemK, pemI, ccdA, ccdB
UMB7780	Enterococcus faecalis			
UMB0843	Enterococcus faecalis			
UMB7783	Klebsiella pneumoniae	traA, traM, tra_I_1, traD_1, finO_1, traN, finO_2, traI_2, traS. traD_2	ssb (plasmid)	
UMB8492	Klebsiella pneumoniae	traD, traQ, traN, traC, traV, traA, traM, finO	ssb (plasmid)	
UMB7779	Klebsiella pneumoniae	fhO, traI, traD, traQ, traN, traC, traV, traA, traY, traJ, traM		
UMB8493	Staphylococcus epidermidis			yoeB
UMB1227	Staphylococcus epidermidis			
UMB0626	Staphylococcus epidermidis			
UMB0593	Staphylococcus epidermidis			
UMB1201	Staphylococcus epidermidis			
UMB9183	Staphylococcus epidermidis			
UMB0567	Streptococcus anginosus			
UMB8616	Streptococcus anginosus		pre	

## DISCUSSION

An urgent goal in urobiome research is to elucidate the mechanisms that link the urobiota to urinary conditions (1, 8, 9). This includes understanding why species can be associated with both asymptomatic and symptomatic states (65, 66). Plasmid content of urinary species may be a key component underlying urobiota behavior and their effect on urinary health (20, 67). Plasmids are important reservoirs and vectors for genetic content in bacteria populations, including virulence and antibiotic resistance genes (17, 43, 68). In this study, we analyzed the plasmid content of representative strains of 11 highly relevant urinary species, broadly grouped in three categories: (i) urinary species in which we could consistently detect plasmid presence and profile gene content (E. coli, K. pneumoniae), (ii) urinary species in which we could detect putative plasmids but most of their content was not profiled via PlasmidFinder or BLAST (E. faecalis, S. anginosus, S. epidermidis), and (ii) urinary species where plasmid presence was negative or inconclusive (A. urinae, C. amycolatum, G. vaginalis, L. gasseri, L. jensenii, S. mitis).

Six of nine urinary E. coli isolates had evidence of at least one plasmid, with an average 31.78% of ORFs assigned a known function (Tables 1–4). E. coli plasmids are some of the best characterized, with F plasmids being especially relevant due to their prevalence, persistence, and transmission of clinically relevant traits, such as antibiotic resistance and virulence factors (15, 20). There is evidence that F plasmids of urinary E. coli are more often found in E. coli linked to UTI in kidney-transplanted patients relative to control (41). The *incX1*, *incB/O/K/Z*, and *col4400I* loci were also found in the E. coli plasmidic assemblies. IncX and IncB/O/K/Z plasmids have been associated with antibiotic resistance, while colicin plasmids are utilized in competition between similar Gram-negative species (69–72). Broadly speaking, plasmids were readily identified in the urinary E. coli isolates either by the presence of replicon loci or homology to plasmid entries in the NCBI nr/nt database. This is likely because of the well-developed plasmid reference databases and assembly algorithms compatible with E. coli’s plasmid genetic content (73, 74). Despite these well-developed databases, only about a third of the predicted ORFs were assigned a function.

Profiling of plasmid content in K. pneumoniae was successful, likely because of its genetic similarity to E. coli (44, 75). The three K. pneumoniae putative plasmids had an average 39.19% of ORFs assigned a known function (Tables 1, 3, and 4). All three putative K. pneumoniae plasmids had high query coverage and sequence identity (>90%) to annotated complete plasmid records in GenBank (Table 3). The *inc* loci present in K. pneumoniae are the Klebsiella variants of Col, IncFIB, and IncFII, which bear similarity to *inc* loci in E. coli and are respectively linked to colicin and F plasmids (76). As stated before, F plasmids are clinically relevant due to their antibiotic resistant and virulent genetic content (20). The presence of colicin plasmids could point to competition between K. pneumoniae and other bacteria species, including E. coli (71, 72). Antibiotic resistance genes were not predicted in K. pneumoniae plasmidic assemblies, but the virulence gene *traT* was predicted in two of them (Table 1). The plasmidic assembly from UMB7779 had 80% sequence query coverage and 100% identity to the plasmid pKpn3-L132 (CP027614.1) present in K. pneumoniae OXA-48, which contributed to a nosocomial outbreak in Taiwan (75).

Plasmids were predicted in six of nine S. epidermidis isolates, with an average 41.43% of ORFs assigned a known function (the highest species average) (Tables 1, 3, and 4). Most of these plasmidic assemblies had high sequence identity but low query coverage with their closest plasmid homolog in GenBank (Table 3). Antibiotic resistance was predicted in these plasmidic assemblies (Table 1), which could make them clinically relevant (50). As an example, the plasmidic assembly from UMB1227 has homology to a conjugative plasmid in clinical S. epidermidis from Italy with multiple resistances (77).

E. faecalis and S. anginosus had plasmidic assemblies that matched plasmid entries in the NCBI nr/nt database, but the tools we employed did not profile genes to the same extent as the three aforementioned species (Tables 1 and 3). The two E. faecalis plasmidic assemblies had an average 22.7% of ORFs assigned a known function (Table 4). Neither E. faecalis plasmidic assembly had evidence of antibiotic resistance genes, but virulence genes were predicted (Table 1). The two S. anginosus isolates with plasmidic assemblies, UMB0567 and UMB8616, had relatively small putative plasmid sequences (5k–6k bp) with only a small number of ORFs predicted and annotated with a function (Tables 1, 3, and 4). These plasmidic assemblies did not have predicted genes for any other plasmid content profiled. The evidence that these isolates have a plasmid relies on their plasmidic assemblies being similar to pDRPIS7493 and paSTHERMO (over 99% identity and respectively 32% and 85% sequence query coverage) (Table 3). However, as *rep* loci were not detected, they may be novel, or these loci were not included in the assembly. Given the lack of replicon loci and relatively low sequence query coverage when compared to known plasmids, further studies are needed to verify that E. faecalis UMB7780 and S. epidermidis UMB0567 do contain a plasmid.

plasmidSPAdes did not produce a plasmidic assembly for any of the urinary isolates considered here for the species A. urinae, G. vaginalis, and S. mitis (Table S1). The remaining urinary species (C. amycolatum, L. gasseri, L. jensenii) had no convincing evidence of plasmid presence, given the absence of replicon loci and very low homology to known plasmids (Table S1 and S2). The short plasmid-like sequences in C. amycolatum, L. gasseri, and L. jensenii could be due to plasmid-like regions in the chromosome of these isolates (e.g., prophage, past genetic exchange with plasmids). However, the absence of evidence is not evidence of absence. There are few studies on the plasmids of these species, which means few references in the databases for comparison (73, 78, 79) and other sequencing methods are required to identify novel plasmids, for example hybrid assembly. Another factor to consider is the relatively small sample size per species analyzed.

We utilized homologous plasmids in Table 3 as a reference to map raw sequence reads from the respective urinary isolate (Table S3). In most cases, a plasmidic assembly was produced that when compared to its reference had a sequence query coverage and identity over 90%. This provides evidence that the plasmid genetic content in these urinary isolates is similar to known plasmid sequences. However, there is a drawback in relying on this method for plasmid assembly, as it limits output to what is already known. Urinary plasmids may contain novel genetic content that is not present in the plasmid reference sequence.

Another important element to consider in plasmid research is the ability of certain plasmids to transfer within and between species (14). Plasmid conjugation genes were identified in plasmidic assemblies from E. coli and K. pneumoniae, specifically *tra* and *virB-virD* genes (Table 5) (80, 81). Potentially relevant is that some plasmidic assemblies from E. coli are from the same Inc groups as those in K. pneumoniae (specifically loci found in F and colicin plasmids) (42, 71). Plasmid exchange is known to occur in E. coli and other Gram-negative species, including Klebsiella, and exchange could be occurring in the urinary tract (44, 81). *traT* also was identified, which aids plasmids in blocking invasion by similar plasmids, suggesting that plasmid competition occurs in the urinary tract for these species (60, 63) (Table 1). Conjugation genes were not identified in E. faecalis, S. epidermidis, or S. anginosus, which begs the following question: Is conjugation simply not present? (77, 82) Alternatively, do these species utilize a system absent in the gene reference database or one that has not been assigned that functionality? Outside the urinary tract, there is evidence that Enterococci can conjugate plasmids, while species of Staphylococci may also rely on transformation and vertical transmission in addition to conjugation (17, 82, 83). Horizontal exchange of plasmids in these urinary species may necessitate the creation of custom reference databases with conjugation and competence genes for appropriate gene profiling.

In this project, we relied on two types of tools: (i) reference-independent (*de novo*) assembly of plasmidic raw sequencing reads based on algorithmic assumptions (i.e., plasmidSPAdes assembly), and (ii) reference-dependent gene profiling (web BLAST, PlasmidFinder, ResFinder, VirulenceFinder, Prokka) (73, 78, 79, 84–86). For plasmidSPAdes, the assembly algorithm has been reported to assemble plasmids in Enterobacteriaceae (E. coli, K. pneumoniae) and Staphylococcaceae (S. epidermidis) (74); employing long-read sequencing for 3 of the E. coli isolates confirmed the capabilities of the plasmidSPAdes tool to properly assemble and identify E. coli plasmids (Table 2). We anticipated that plasmid assembly would occur in E. faecalis, S. anginosus, and S. mitis given their genetic overlap with Staphylococci. More unpredictable was plasmid assembly in the other species (A. urinae, C. amycolatum, G. vaginalis, L. gasseri, L. jensenii), where less is known of their plasmid size, circular/linear composition, and copy number, especially in the urinary tract (54, 58, 59). Consequently, it is in the latter species where we saw either no or inconclusive plasmid results. The results of the reference-dependent method were more predictable, however. Reference-based profiling relies on a robust database so that queries can have matches (84, 86). The databases we utilized are primarily composed of well-studied organisms, such as E. coli and Staphylococcus species (78, 79, 85). In urinary research, species such as those in the genera Aerococcus, Corynebacterium, Gardnerella, and Lactobacillus are understudied, and much of their genetic content is unknown (23, 26).

A limitation of this study was the use of plasmidSPAdes plasmidic assemblies as representatives of plasmids in these urinary species. While not perfectly accurate, plasmidic assemblies are still a valid representation of plasmid content in bacteria (73, 74). Three of the four plasmidic assemblies produced by plasmidSPAdes were able to be confirmed and closed via long-read sequencing; the fourth, one of the UMB1284 short-read plasmidic assemblies, was identified by long-read sequencing, but the long-read contig was significantly longer than the one produced by plasmidSPAdes (Table 2). Further investigation into this particular strain is needed to ascertain the structure of this plasmid. Initial studies, such as this one, are necessary to build a more complete understanding of plasmids in the urinary tract.

In terms of future projects that study plasmid content in urinary species, we envision two broad strategies. For well-studied bacteria like those from the families Enterobacteriaceae and Staphylococcaceae, it is feasible to organize high-throughput genomic profiling, even from existing WGS raw short reads. This can be streamlined by scripting a pipeline of commonly used assemblers (e.g., plasmidSPAdes) then scanning the output with reference databases (e.g., Prokka, local BLAST) (73, 84, 86). While many urinary E. coli and K. pneumoniae strains are publicly available, we included just a few here serving as a control, given the wealth of information available for their plasmids and the fact that many tools/databases for plasmid bioinformatics have been benchmarked using E. coli. Bacteria from the families Enterococcaceae and Streptococcaceae face more challenges in terms of specific gene profiling, but it is realistic to pinpoint whether plasmids are present in these species with the approach we employed, especially if custom reference databases are utilized. Finally, for less studied bacterial genera (e.g., Aerococcus, Corynebacterium, Gardnerella, Lactobacillus), a more arduous approach is required for predicting plasmid presence and profiling genetic content. Our recommendation would be to rely on known plasmid sequences in members of these species isolated from outside the urinary tract to predict presence of plasmids in urinary isolates. Using this strategy, the impetus would be on building reference databases specific to a family or genus. Long-read sequencing of these genera, both from the urobiome and elsewhere, may aid in building this reference database.

In this study, we employed genomics to analyze the presence of plasmids in urinary isolates of 11 bacteria species relevant to urinary health. The contribution of plasmids to urinary health have not been thoroughly assessed, even in species in the families Enterobacteriaceae and Staphylococcaceae, which are arguably the best studied bacteria in microbiology (1, 7, 9). Plasmids have immense clinical and biological relevance, able to retain and transmit key traits like antibiotic resistance and virulence (15, 20). Plasmid research could shed light into the important question of why isolates from many of these species can be present in people that can be both symptomatic and asymptomatic for urinary conditions (65, 66). This study provides a starting point for plasmid content in urobiota species linked to LUTS and brings attention to the need for wet lab research and specialized bioinformatic tools to further characterize bacterial plasmids in the urinary tract.

## MATERIALS AND METHODS

The draft genomes of 71 urinary isolates from 11 species (A. urinae (N = 7), C. amycolatum (N = 5), E. coli (N = 9), E. faecalis (N = 3), G. vaginalis (N = 5), K. pneumoniae (N = 3), L. gasseri (N = 6), L. jensenii (N = 8), S. epidermidis (N = 9), S. anginosus (N = 8), and S. mitis (N = 8)) were previously sequenced, assembled, and made publicly available by our group (BioProject PRJNA316969) (Table S1). To create plasmid assemblies, raw sequence reads (SRA) were downloaded for these species and assembled using plasmidspades.py of SPAdes v3.12 with k values of 55,77,99,127 and the only-assembler parameter (73). plasmidSPAdes takes the assembly graph from SPAdes and classifies a subgraph as the plasmid graph, which is further processed into plasmidic assemblies (87, 88). “Plasmidic assemblies contain plasmid-like sequences from the WGS, though further curation and pruning may be necessary to remove false positives” (73, 74). In this study we assess plasmidic assemblies as a representative of plasmid content. Assemblies were renamed via a Bash script, and contigs less than 500 bp were removed via bioawk. Each contig in the plasmidic assembly was queried via megablast against the nr/nt database, and contigs with homology to plasmid records were retained for further analysis while contigs with chromosomal homology were removed from further consideration (84). Assemblies were then concatenated as a single read and queried via megablast against the nr/nt database; assemblies were organized on their sequence query coverage, percent identity, and E-value to plasmid entries. The Bowtie2 (version 2.3.2.) plug-in in Geneious Prime v2021.1 was used to verify even coverage of raw sequence reads to the NCBI plasmid record with highest homology to a given plasmid assembly (89). Curated plasmidic assemblies are publicly available through the BioProject and their strain’s respective BioSample Assembly (Assembly Database); the BioSample accession numbers for each strain examined are included in Table S1. Furthermore, plasmidic assembly sequences can be directly accessed via FigShare (https://doi.org/10.6084/m9.figshare.17005318.v1).

To identity *rep* and *inc* loci, the putative plasmid assemblies were scanned with PlasmidFinder v2.1, using either the Enterobacteriaceae or Gram-positive database, with a threshold of 95% identity and a minimum 60% coverage (85). To identify known antibiotic resistance genes, the FASTA files were scanned with ResFinder v4.1 using the “acquired antimicrobial resistance genes” option (78). To identify known virulence genes, the FASTA files were scanned with VirulenceFinder v2.0 with an identity threshold of 90%, and the “minimum sequence length of 60%” option (79). Plasmid assemblies were annotated using Prokka v1.14.5 with default parameters in addition to parameters -entre XXX and -compliant (86). Annotation output files were renamed and reorganized using a Bash script. The annotated ORFs were reviewed for predicted functions related to plasmid transfer, replication, and addiction/retention.

E. coli isolates UMB1180, UMB1195, and UMB1284 were sequenced using Nanopore long-reads sequencing. Each strain was streaked from cultures stored at −80°C onto colistin nalidixic acid (CAN) plates and incubated overnight at 35°C with 5% CO_2_. Single colonies were selected and grown in liquid LB overnight at 37°C, with shaking. DNA was extracted using the QIAmp DNA minikit with the following exceptions: samples were not vortexed, rather agitated by hand, and the lysis step was conducted for 1.5–2 h at 37°C. DNA was then shipped to the Columbia University Core Facility (New York, NY), where libraries were prepared using the Rapid Barcoding 96 kit (Oxford Nanopore, SQK-RBK110.96) according to the manufacturer’s instructions and sequenced on an Oxford Nanopore GridION using an R9.4.2 flow cell. High accuracy basecalling and demultiplexing was performed using MinKNOW v21.05.20. Read QC was performed using Porechop v0.2.4 (https://github.com/rrwick/Porechop) to remove adapter and barcode sequences and mothur v1.25.0 (https://mothur.org/) to remove long homopolymeric regions (>20 bp) and short reads (<1,000 bp). These filtered reads were then assembled using Unicycler v0.4.9 with default parameters (90). Nanopore reads have been deposited in SRA, associated with the BioSample Accession Numbers for the three strains (indicated in Table S1).
