# Supplementary material for: Characterizing Plasmids in Bacteria Species Relevant to Urinary Health
Source: Microbiol Spectr. 2021 Dec 22;9(3):e00942-21. doi: 10.1128/spectrum.00942-21 (PMC8694116; doi:10.1128/spectrum.00942-21)
Supplement: SUPPLEMENTAL FILE 1 — Supplemental material. Download SPECTRUM00942-21_Supp_1_seq5.pdf, PDF file, 0.2 MB [file spectrum00942-21_supp_1_seq5.pdf]

Supplemental Table 1. Genomes of urinary species analyzed in this study

Strain	Species	BioSample Accession #	Individual Information	plasmidSPAdes Assembly?	Plasmid replicon?	Plasmidic assembly homology to plasmids?
UMB637	*Aerococcus urinae*	SAMN12797010	Female, catheterized urine, OAB	No	No	No
UMB970	*Aerococcus urinae*	SAMN12797012	Female, catheterized urine, SUI	No	No	No
UMB2126	*Aerococcus urinae*	SAMN12797023	Female, catheterized urine, OAB	No	No	No
UMB7049	*Aerococcus urinae*	SAMN12797031	Female, catheterized urine, OAB	No	No	No
UMB8614	*Aerococcus urinae*	SAMN12797038	Female, catheterized urine, OAB	No	No	No
UMB8662	*Aerococcus urinae*	SAMN12797041	Female, catheterized urine, OAB	No	No	No
UMB8711	*Aerococcus urinae*	SAMN12797043	Female, catheterized urine, OAB	No	No	No
UMB9184	*Corynebacterium amycolatum*	SAMN12797046	Female, catheterized urine, rUTI	Yes	No	Yes
UMB9256	*Corynebacterium amycolatum*	SAMN12797048	Female, catheterized urine, UTI	No	No	No
UMB1182	*Corynebacterium amycolatum*	SAMN12797015	Female, catheterized urine, UTI	No	No	No
UMB1310	*Corynebacterium amycolatum*	SAMN12797022	Female, catheterized urine, UTI	Yes	No	Yes
UMB7760	*Corynebacterium amycolatum*	SAMN12797034	Female, catheterized urine, rUTI	Yes	No	Yes
UMB0843	*Enterococcus faecalis*	SAMN14478480	Female, catheterized urine, pregnant	Yes	No	Yes
UMB1309	*Enterococcus faecalis*	SAMN14478485	Female, catheterized urine, UTI	Yes	No	Yes
UMB7780	*Enterococcus faecalis*	SAMN14478493	Female, catheterized urine, rUTI	Yes	No	Yes
UMB1180	*Escherichia coli*	SAMN09665180	Female, catheterized urine, UTI	Yes	Yes	Yes
UMB1195	*Escherichia coli*	SAMN09665182	Female, catheterized urine, UTI	Yes	Yes	Yes
UMB1225	*Escherichia coli*	SAMN09665186	Female, catheterized urine, UTI	No	No	No
UMB1284	*Escherichia coli*	SAMN09665189	Female, catheterized urine, UTI	Yes	Yes	Yes
UMB1353	*Escherichia coli*	SAMN14478487	Female, catheterized urine, UTI	No	No	No
UMB7764	*Escherichia coli*	SAMN12797035	Female, catheterized urine, rUTI	Yes	Yes	Yes
UMB9182	*Escherichia coli*	SAMN12797044	Female, catheterized urine, rUTI	Yes	No	Yes
UMB9246	*Escherichia coli*	SAMN14478501	Female, catheterized urine, rUTI	Yes	Yes	Yes
UMB9250	*Escherichia coli*	SAMN12797047	Female, catheterized urine, UTI	Yes	Yes	Yes

Strain	Species	BioSample Accession #	Individual Information	plasmidSPAdes Assembly?	Plasmid replicon?	Plasmidic assembly homology to plasmids?
UMB0170	*Gardnerella vaginalis*	SAMN07511406	Female, catheterized urine, OAB	No	No	No
UMB0264	*Gardnerella vaginalis*	SAMN07511407	Female, catheterized urine, OAB	No	No	No
UMB0768	*Gardnerella vaginalis*	SAMN07511408	Female, catheterized urine, no LUTS	No	No	No
UMB1642	*Gardnerella vaginalis*	SAMN07511411	Female, catheterized urine, no LUTS	No	No	No
UMB1686	*Gardnerella vaginalis*	SAMN07511412	Female, catheterized urine, diabetes	No	No	No
UMB7779	*Klebsiella pneumoniae*	SAMN14478492	Female, catheterized urine, rUTI	Yes	Yes	Yes
UMB7783	*Klebsiella pneumoniae*	SAMN14478495	Female, catheterized urine, rUTI	Yes	Yes	Yes
UMB8492	*Klebsiella pneumoniae*	SAMN14478498	Female, catheterized urine, OAB	Yes	No	Yes
UMB0607	*Lactobacillus gasseri*	SAMN12277452	Female, catheterized urine, SUI	Yes	No	Yes
UMB1196	*Lactobacillus gasseri*	SAMN12277456	Female, catheterized urine, UTI	Yes	No	Yes
UMB1399	*Lactobacillus gasseri*	SAMN12277447	Female, catheterized urine, no LUTS	No	No	No
UMB2965	*Lactobacillus gasseri*	SAMN12277457	male, catheterized urine, OAB	No	No	No
UMB3077	*Lactobacillus gasseri*	SAMN12277448	male, catheterized urine, OAB	No	No	No
UMB4205	*Lactobacillus gasseri*	SAMN12277458	male, catheterized urine, OAB	No	No	No
UMB0034	*Lactobacillus jensenii*	SAMN12277449	Female, catheterized urine, no LUTS	No	No	No
UMB0037	*Lactobacillus jensenii*	SAMN12277450	Female, catheterized urine, no LUTS	No	No	Yes
UMB0055	*Lactobacillus jensenii*	SAMN12277451	Female, catheterized urine, no LUTS	No	No	No
UMB0732	*Lactobacillus jensenii*	SAMN12277459	Female, catheterized urine, no LUTS	Yes	No	Yes
UMB1165	*Lactobacillus jensenii*	SAMN12277443	Female, catheterized urine, UTI	No	No	No
UMB1307	*Lactobacillus jensenii*	SAMN12277444	Female, catheterized urine, UTI	No	No	No
UMB1355	*Lactobacillus jensenii*	SAMN12277446	Female, catheterized urine, UTI	Yes	No	Yes
UMB8489	*Lactobacillus jensenii*	SAMN12277454	Female, catheterized urine, OAB	No	No	No
UMB593	*Staphylococcus epidermidis*	SAMN12797002	Female, catheterized urine, SUI	Yes	No	Yes
UMB626	*Staphylococcus epidermidis*	SAMN12797008	Female, catheterized urine, SUI	Yes	No	Yes
UMB1201	*Staphylococcus epidermidis*	SAMN12797018	Female, catheterized urine, UTI	Yes	No	Yes
UMB1227	*Staphylococcus epidermidis*	SAMN12797020	Female, catheterized urine, UTI	Yes	Yes	Yes

Strain	Species	BioSample Accession #	Individual Information	plasmidSPAdes Assembly?	Plasmid replicon?	Plasmidic assembly homology to plasmids?
UMB1188	*Staphylococcus epidermidis*	SAMN12797016	Female, catheterized urine, UTI	No	No	No
UMB7759	*Staphylococcus epidermidis*	SAMN12797033	Female, catheterized urine, rUTI	No	No	No
UMB7765	*Staphylococcus epidermidis*	SAMN14478488	Female, catheterized urine, rUTI	No	No	No
UMB8493	*Staphylococcus epidermidis*	SAMN14478499	Female, catheterized urine, OAB	Yes	No	Yes
UMB9183	*Staphylococcus epidermidis*	SAMN12797045	Female, catheterized urine, rUTI	Yes	Yes	Yes
UMB248	*Streptococcus anginosus*	SAMN12796999	Female, catheterized urine, OAB	No	No	No
UMB567	*Streptococcus anginosus*	SAMN12797000	Female, catheterized urine, UUI	Yes	No	Yes
UMB0839	*Streptococcus anginosus*	SAMN08193720	Female, catheterized urine, pregnant	No	No	No
UMB1296	*Streptococcus anginosus*	SAMN14478484	Female, catheterized urine, UTI	No	No	No
UMB7768	*Streptococcus anginosus*	SAMN14478490	Female, catheterized urine, rUTI	No	No	No
UMB8390	*Streptococcus anginosus*	SAMN12797036	Female, catheterized urine, OAB	No	No	No
UMB8616	*Streptococcus anginosus*	SAMN12797039	Female, catheterized urine, OAB	Yes	No	Yes
UMB8710	*Streptococcus anginosus*	SAMN12797042	Female, catheterized urine, OAB	Yes	No	Yes
SM17	*Streptococcus mitis*	SAMN13105986	Female, voided urine, no LUTS	No	No	No
SM18	*Streptococcus mitis*	SAMN13105987	Female, voided urine, no LUTS	No	No	No
SM19	*Streptococcus mitis*	SAMN13105988	Female, voided urine, no LUTS	No	No	No
SM36	*Streptococcus mitis*	SAMN13105990	Female, voided urine, no LUTS	No	No	No
SM37	*Streptococcus mitis*	SAMN13105991	Female, voided urine, no LUTS	No	No	No
SM42	*Streptococcus mitis*	SAMN13105992	Female, voided urine, no LUTS	No	No	No
SM49	*Streptococcus mitis*	SAMN13105984	Male, oral swab	No	No	No
SM50	*Streptococcus mitis*	SAMN13105985	Male, oral swab	No	No	No

UTI=Urinary Tract Infection; rUTI=Recurrent UTI; OAB=Overactive Bladder Symptoms; UUI=Urge Urinary Incontinence; SUI=Stress Urinary Incontinence; no LUTS=No Lower Urinary Tract Symptoms (”healthy” or “asymptomatic”)

Supplemental Table 2. Plasmidic assemblies with low plasmid homology

Strain	Species	Assembly size	NCBI database plasmid entry	Max Score	Total Score	Sequence query Coverage	E value	Per. Ident	Acc. Len	Accession	Plasmid size (bp)
UMB9184	*Corynebacterium amycolatum*	42723	phiCFAL8171	632	632	3%	5.00E-175	75.03%	42009	CP007157.1	42009
UMB1310	*Corynebacterium amycolatum*	19581	pFb21	62.1	62.1	0%	0.001	87.27%	606697	CP034941.1	606697
UMB1196	*Lactobacillus gasseri*	651856	pJV-V03	4047	4109	1%	0	96.71%	41709	CP040501.1	41709
UMB0607	*Lactobacillus gasseri*	992128	pJV-V03	4047	4109	1%	0	96.71%	41709	CP040501.1	41709
UMB0037	*Lactobacillus jensenii*	228609	p1	309	633	0%	7.00E-78	92.99%	33688	CP002612.1	33688
UMB1355	*Lactobacillus jensenii*	218414	p1	1646	2331	3%	0	97.32%	33688	CP002612.1	33688
UMB0732	*Lactobacillus jensenii*	331642	p1	309	633	0%	7.00E-78	92.99%	33688	CP002612.1	33688
UMB8710	*Streptococcus anginosus*	33727	pARO1.4	106	106	3%	6.00E-18	95.45%	5121	CP022487.1	5121

Supplemental Table 3. Web BLAST summary of urinary plasmidic assemblies constructed by reference mapping

Strain	Species	Urinary plasmid length (bp)	Plasmid hit	Query Coverage	E value	Per. Ident	Length (bp)	Accession
UMB7780	*Enterococcus faecalis*	24490	pEcl5-3	99%	0	99.98%	4863	CP047739.1
UMB0843	*Enterococcus faecalis*	46433	pFDAARGOS_324	99%	0	99.03%	49440	CP028283.1
UMB1309	*Enterococcus faecalis*	25133	pAUSMDU00004142	88%	0	96.39%	168778	CP027502.1
UMB9250	*Escherichia coli*	27648	p2012C-4431	98%	0	98.69%	36473	CP024290.1
UMB1195	*Escherichia coli*	113976	pRHBSTW-00152_2	99%	0	99.86%	133729	CP056811.1
UMB1284	*Escherichia coli*	73399	pECAZ162_KPC	91%	0	100.00%	142829	CP019014.1
UMB1284_1	*Escherichia coli*	33879	p51008369SK1_C	99%	0	99.89%	33826	CP029976.1
UMB1180	*Escherichia coli*	4877	pRHBSTW-00152_2	99%	0	99.86%	133729	CP056811.1
UMB9182	*Escherichia coli*	80734	p009_C	99%	0	95.81%	94239	CP048307.1
UMB7764	*Escherichia coli*	46499	p1658/97	98%	0	99.91%	125491	AF550679.1
UMB9246	*Escherichia coli*	77162	pSCU-313-1	99%	0	99.90%	105394	CP051695.1
UMB7783	*Klebsiella pneumoniae*	100769	pAR_0096	99%	0	99.98%	100759	CP027614.1
UMB8492	*Klebsiella pneumoniae*	100773	pAR_0096	99%	0	99.93%	100759	CP027614.1
UMB7779	*Klebsiella pneumoniae*	170153	p1_020049	99%	0	99.78%	182097	CP028784.1
UMB8493	*Staphylococcus epidermidis*	34575	pSESURV_p3_1362	98%	0	98.49%	54404	CP043803.1
UMB1227	*Staphylococcus epidermidis*	35173	pSP01	99%	0	99.89%	76991	KR230047.1
UMB626	*Staphylococcus epidermidis*	21273	pFDAARGOS_161	99%	0	99.66%	21267	CP014130.1
UMB593	*Staphylococcus epidermidis*	4264	pt248	93%	0	98.32%	28396	CP049450.1
UMB1201	*Staphylococcus epidermidis*	22612	p16A	97%	0	94.80%	108569	CP031267.1
UMB9183	*Staphylococcus epidermidis*	11656	pFDAARGOS_913	99%	0	99.74%	11858	CP065654.1
UMB567	*Streptococcus anginosus*	2047	pDRPIS7493	92%	0	97.65%	4727	CP002926.1
UMB8616	*Streptococcus anginosus*	4320	paSTHERMO	97%	0	96.80%	4451	LR822024.1
